# A self-regulation intervention conducted by class teachers: impact on elementary students’ basic psychological needs and classroom engagement

**DOI:** 10.3389/fpsyg.2023.1220536

**Published:** 2023-11-23

**Authors:** Jennifer Cunha, Juliana Martins, Rafaela Peseta, Pedro Rosário

**Affiliations:** Psychology Research Centre, School of Psychology, University of Minho, Braga, Portugal

**Keywords:** basic psychological needs, classroom engagement, elementary school, narrative-based intervention, self-regulated learning

## Abstract

Literature has reported a decrease in students’ engagement throughout schooling, but more worrying, is that elementary students already show signs of disengagement. This data sets the case to develop interventions at this school level. The narrative-based intervention “Yellow Trials and Tribulations” aimed to promote self-regulation has been proven to positively impact elementary students’ school engagement. Acknowledging that classroom engagement is expected to be more closely related to learning and achievement than school engagement, the current study aims to extend the research of the mentioned intervention on elementary students’ classroom engagement (i.e., behavioral, emotional, cognitive, and agentic dimensions), as well as on basic psychological needs (i.e., perceived autonomy, competence, and relatedness)—an antecedent of students’ engagement. The current intervention was implemented by 4th-grade class teachers trained for that purpose and was assessed following a quasi-experimental design with pretest and posttest data collection. Participants were 90 students in the experimental group, and 91 in the comparison group. A multivariate analysis of variance with repeated measures was run for each construct. At the end of the intervention, children in the experimental group reported higher perceived competence and classroom engagement (all dimensions) than their counterparts in the comparison group (small and medium effect sizes were found). No follow-up was conducted to examine whether the intervention effects were long-lasting. Results are expected to support researchers’ and educators’ efforts to effectively implement the intervention, and maximize its benefits to students. For example, extra efforts could be made to help implementers better respond to students’ psychological needs (in this case, perceived autonomy and relatedness), and consequently increase classroom engagement (especially behavioral and emotional engagement, which revealed lower effect sizes).

## 1 Introduction

Socio-Emotional Learning (SEL) involves a wide range of skills (e.g., self-regulation of emotions, behaviors, and thoughts), with an important role in students’ academic learning while facilitating students’ engagement and school success (e.g., Cristóvão et al., 2017; Sala et al., 2020). Students’ engagement is an important indicator of students’ motivation and learning in elementary school, and later in high school (Côté-Lussier and Fitzpatrick, 2016; Estévez et al., 2021). However, recent research reports early signs of low engagement at the elementary school level, which may compromise subsequent learning and academic trajectories (e.g., Archambault and Dupéré, 2017). This data sets the case to develop interventions on this topic as soon as possible to prevent students from falling into a maladaptive academic trajectory (Luo et al., 2009; Skinner et al., 2016). For the purpose of this research; following Reeve (2012), student engagement is defined as students’ active involvement in a learning activity, which involves four dimensions as follows: behavioral, emotional, cognitive, and agentic engagement (see the section Engagement: definition and empirical evidence).

Literature provides several examples of school-based interventions with distinct natures, despite all being focused on promoting elementary students’ engagement (e.g., academic tasks, behavior monitoring; see Martins et al., 2021). Of the existing interventions, the narrative-based intervention “Yellow Trails and Tribulations” developed by Rosário et al. (2007a,b) aimed to promote self-regulation skills. This intervention has been shown to positively impact students’ school engagement (Rosário et al., 2016; Azevedo et al., 2023); however, literature alerts that not all types of school engagement contribute equally to learning and achievement (Skinner and Pitzer, 2012). For example, students’ engagement while completing academic tasks is more likely to impact students’ academic learning than their engagement in general school activities or initiatives (Skinner et al., 2009). Prior studies using the “Yellow Trails and Tribulations” intervention did not differentiate components of school and classroom engagement (see Rosário et al., 2016; Azevedo et al., 2023), which may have prevented the retrieval of pertinent information to improve specific aspects of students’ learning. Moreover, recent qualitative data indicates that students who participated in the narrative-based intervention were perceived by their teachers as being more confident and engaged in class, even the students with low prior achievement (Cunha et al., 2023). This data, despite being limited to teachers’ and observers’ overall perceptions of the intervention impact, led us to hypothesize that the “Yellow Trails and Tribulations” narrative-based intervention may contribute to satisfying students’ psychological needs and increasing classroom engagement. In this context, the current study aims to extend our knowledge of the benefits of this intervention.

Anchored on Self-Determination Theory, the current study examined the impact of the intervention “Yellow Trials and Tribulations” on elementary students’ motivational variables (i.e., basic psychological needs satisfaction of perceived autonomy, competence and relatedness) and classroom engagement, through the training of self-regulated learning skills, a component of socio-emotional core skills. Findings are expected to provide granular information on the impact of the intervention, and implications for effective educational practice.

### 1.1 Theoretical framework of the study

Self-determination Theory (SDT) provides a relevant theoretical framework for the current study. The Basic Psychological Needs Theory is one of the SDT’s six mini theories (Deci and Ryan, 2000; Ryan and Deci, 2020). This mini theory postulates perceived autonomy, competence, and relatedness as basic psychological needs universal and innate to individuals (Deci and Ryan, 2000). Autonomy refers to the individual’s ability to be responsible for their behaviors while self-regulating them toward an internal locus of causality (e.g., students’ willingness to dedicate time and energy to study; Deci and Ryan, 2000; Niemiec and Ryan, 2009). Autonomy is likely to be satisfied when individuals experience choice over their actions, enthusiasm, and appreciation (Skinner and Belmont, 1993; Ryan and Deci, 2020). Perceived competence relates to individuals’ ability to perform meaningful assignments in a specific context and experience mastery while completing an academic task (e.g., Deci and Ryan, 2000; Conesa and Duñabeitia, 2021). Individuals who experience positive feedback are likely to satisfy their need for competence (Skinner and Belmont, 1993; Ryan and Deci, 2020). Lastly, relatedness describes the need to create meaningful relations and to connect with others (e.g., quality of the relationship with teachers and peers in the classroom; Skinner and Belmont, 1993; Deci and Ryan, 2000). This need is likely to be satisfied when individuals experience a sense of belongingness, respect, and security (e.g., students who feel that teachers genuinely value and respect their work; Van den Broeck et al., 2016; Ryan and Deci, 2020).

According to literature, this theory advances with a deep and integrated explanation of student functioning, and helps to explain the role of (dis)satisfaction of basic psychological needs as an underlying process of (dis)engagement during learning activities (Deci and Ryan, 2000; Jang et al., 2012, 2016; Reeve, 2012). Students must fulfill their basic psychological needs in order to learn, and function positively in the classroom (e.g., Deci and Ryan, 2000; Reeve, 2012; Reeve and Lee, 2014). SDT sustains that the fulfillment of these basic psychological needs allows an increase in students’ autonomous motivation and engagement and an indirect enhancement of academic achievement (Jang et al., 2012). As prior research found, students who fulfill their basic psychological needs in class are more likely to engage in their school learning (Hughes et al., 2008; Niemiec and Ryan, 2009; Reeve, 2012; Schuitema et al., 2016), which positively influences their willingness to acquire knowledge, develop socially and cognitively, experience gratification, and progress in schooling (e.g., Marks, 2000; McClelland et al., 2006).

### 1.2 Engagement: definition and measures

Student engagement has been studied by researchers and educators for more than three decades (e.g., Martins et al., 2021). This is a multidimensional construct, co-existing various definitions and dimensions at different levels (e.g., school, classroom, curriculum-based activities), which are nested within each other (see Fredricks and McColskey, 2012; Skinner and Pitzer, 2012; Martins et al., 2021). For example, student engagement in school (or simply school engagement), according to Fredricks et al. (2004)—whose conceptualization has reached more consensus among the literature on the topic (see Martins et al., 2021)—is conceptualized as a three-arm construct encompassing three dimensions: behavioral (e.g., attendance, participation in school activities, effort while forming class activities, doing homework), emotional (i.e., identification and belongingness with school, positive emotional reactions toward school activities, teachers and peers), and cognitive (i.e., students investment in academic activities, use of self-regulatory strategies). All three dimensions comprise indicators of students’ engagement in and out of the school. This general level of engagement is essential to prevent school dropout and promote high school graduation (e.g., Skinner and Pitzer, 2012).

On the other hand, student engagement with learning activities occurring in the classroom context (i.e., a more restricted level of engagement also termed classroom engagement), specifically focuses on the engagement processes occurring in the classroom, such as task-related interactions or whole-class discussions (see Jang et al., 2016). According to Reeve (2012), student engagement may be defined as students’ active involvement in a learning activity and encompasses four dimensions: (i) behavioral engagement which refers to attention, concentration, effort, and persistence when completing a task; (ii) emotional engagement which concerns emotions that help the execution of the task, such as interest, enjoyment, curiosity, and the absence of emotions likely to impair the task such as anger or frustration; (iii) cognitive engagement which refers to the use of learning strategies (e.g., elaboration) and self-regulatory strategies (e.g., planning), and the search for deep conceptual comprehension of the content acquired; and finally, (iv) agentic engagement which refers to the importance of being dynamic, proactive, inquisitive while contributing to the learning process (e.g., asking questions, expressing opinions, and communicating one’s own interests in class discussions). Following Reeve’s (2012) conceptualization of student engagement, the emphasis put in a “learning activity” is crucial to concretely identify the focus or the specific event (i.e., class activities) in which the students are engaged.

The specification of the level of engagement is relevant given the differential impact it may have on students’ educational paths. For example, a student may be engaged in school-related activities (e.g., participating in extracurricular activities), but not in classroom and content-focused activities (and *vice-versa*). As these levels of engagement differ, it is expected that their influence on students’ learning and outcomes would also vary. In this context, Skinner and Pitzer (2012) stated “No matter how many extracurriculars students undertake or how attached they are to school, they will not learn or achieve unless they are constructively engaged with the academic work of the classroom” (pp. 22–23). This means that the level of students’ engagement will somehow determine which students’ outcome variables would be influenced.

Acknowledging engagement as a multidimensional construct encompassing different levels, researchers have been emphasizing the need to measure all dimensions of engagement according to the theoretical framework of the study and focusing on a specific level (e.g., school or classroom) (Wang et al., 2014; Fredricks et al., 2016; Martins et al., 2021). Engagement can be assessed through various methods (e.g., self-reports, observations, school records, interviews, and experience sampling) that may be used as a single method or combined (Fredricks and McColskey, 2012; Azevedo et al., 2023). The ideal procedure would be to combine methods; however, this can be extremely time and resource-consuming. This aspect acquires more relevance when collecting data with large samples (e.g., students of various schools). Self-report measures, despite some limitations, are suited to collect data with large samples, while being a reliable and valid method to measure learning-related internal processes (Pekrun, 2020), which is the case of student engagement in school and in the classroom (Fredricks and McColskey, 2012).

Prior reviews summarized student engagement measures considering the items, dimensions, levels, and samples used (see Fredricks and McColskey, 2012; Martins et al., 2021). Regarding self-report instruments for elementary students, contrary to literature recommendations, several instruments encompass items of the school and classroom levels (e.g., Student Engagement Instrument, Appleton et al., 2006; School Engagement Measure, Fredricks et al., 2005) or assess just one or two dimensions of classroom engagement (e.g., Engagement vs. Disaffection with Learning, Skinner et al., 2009; Eight-item scale assessing children’s classroom engagement behaviors; Pagani et al., 2010). Future studies are expected to overcome these inconsistencies by following a solid theoretical framework and coherently selecting a multidimensional measure of a specific level of engagement.

### 1.3 How to promote students’ engagement?

Acknowledging the importance of engagement and its implications in students’ academic path, researchers put their efforts in identifying students’ characteristics as well as facilitators (i.e., parents, teachers, peers), practices, and optimal contexts for the promotion of students’ engagement in elementary school (see Martins et al., 2021). Not disregarding the importance and the existence of multiple and simultaneous sources of influence (e.g., parents, teachers, peers), prior studies on elementary school have mainly focused on aspects associated to the school environment (e.g., context characteristics) and related micro aspects (e.g., teacher-student relationships and interactions; teachers’ practices in class; school-based interventions) to assess its impact on students’ engagement (Martins et al., 2021). However, it is important to note that a significant number of the studies addressing engagement have a noninterventional nature, intending to test theories or map relationships between student’s and school’s variables and engagement (e.g., Hulleman and Barron, 2016; Pino-James et al., 2019). Despite contributing to improve learning about the construct and allowing to draw educational implications for practice; *per se* these noninterventional studies, “do not end up changing practice” (Hulleman and Barron, 2016). In this context, intervention programs emerge as a suited response to promote students’ engagement while purposefully implementing some changes in the school setting and class dynamics (Lazowski and Hulleman, 2015). As Lazowski and Hulleman (2015) stated, through classroom interventions, an agent (usually a teacher or researcher) has the opportunity to act intentionally and foster change in students’ behaviors, emotions and cognitions in class. According to the literature (see Fredricks et al., 2019; Martins et al., 2021) a considerable number of interventions have been conducted in classrooms to promote student engagement. These interventions with different purposes, address diverse variables (e.g., academic tasks, reading comprehension, behavior monitoring, and teachers’ evidenced-based practices), and were delivered in distinct modalities (e.g., in-class instruction, Mullender-Wijnsma et al., 2015; after school schedule as extra support, Rosário et al., 2016; or in contexts other than schools; Rosário et al., 2017b). Notwithstanding the interventions’ specificities, all reported to positively influence some or all dimensions of engagement (Martins et al., 2021). Therefore, school intervention programs (and studies) to promote students’ engagement are of great importance. Reasons are twofold. School-based intervention programs (i) allow the selection of relevant facilitators of students’ engagement—teachers playing the implementer role (Ryan and Deci, 2020); and (ii) can lead educators and researchers to be one step closer in identifying potential effective educational practices (e.g., suggested in prior empirical studies) and assessing their suitability in meeting students’ educational needs (e.g., Pino-James et al., 2019).

Previous studies have also reported the relevance of promoting students’ engagement through the training of self-regulation processes (e.g., Fitzpatrick, 2012; Rosário et al., 2016; Azevedo et al., 2023; Martins et al., 2023). According to Zimmerman’s (2002) model, self-regulation is a multidimensional construct that refers to the individual’s efforts to orchestrate feelings, thoughts, and actions displayed to attain self-set goals. To learn class content and engage in class, students are expected to not only use a set of cognitive strategies (e.g., working memory or problem-solving strategies), but also to be able to focus their attention and inhibit disruptive behaviors, overcoming background constraints (Fitzpatrick, 2012). The use of these strategies and skills as tools to attain goals involves self-regulation and the exercise of willful control over behavior (Fitzpatrick, 2012; Archambault and Dupéré, 2017; Pereira et al., 2021).

### 1.4 Engagement and self-regulation

Engagement and self-regulation are distinct but intertwined constructs, sharing some characteristics and processes (e.g., students’ involvement, focus and participation in academic-related tasks) implicated in students learning (Ben-Eliyahu et al., 2018; Stefansson et al., 2018). Despite being related, both play an independent but complementary role in the promotion of students’ effective learning (Cleary and Zimmerman, 2012). According to literature (e.g., Reeve and Tseng, 2011; Ben-Eliyahu et al., 2018), the use of self-regulation learning (SRL) strategies presupposes the existence of some degree of engagement. In other words, to self-regulate their learning, students should be minimally engaged in learning activities (e.g., Reeve and Tseng, 2011) otherwise they would not put any effort into their performance. Therefore, the training on self-regulation may contribute to facilitating students’ classroom engagement in a way that while applying behavioral, emotional, and cognitive efforts in classroom tasks, students are simultaneously engaging in these tasks in an active and productive way (Reeve and Tseng, 2011; Ben-Eliyahu et al., 2018). Grounded in this knowledge, providing students with training in SRL strategies seems to be a suitable way to promote the fulfillment of students’ basic psychological needs, and engagement.

### 1.5 Purpose of the study

Elementary school is a critical developmental period for students’ learning because students are expected to learn basic skills (e.g., reading and math; Hill et al., 2008) and acquire essential knowledge to ground future learning experiences (Reyna and Brainerd, 2007). In the Portuguese educational system, fourth grade is the last year of elementary school and sets the ground for the transition to middle school. In the Portuguese middle school (fifth to ninth grade), students have 10 subjects with different teachers, the class size increases, the workload is heavier (e.g., more homework assignments), and finally, students are expected to engage in increased autonomous study time (Cleary and Zimmerman, 2004; Wang and Hofkens, 2020; Santos et al., 2021). The transition from elementary to middle school can be challenging for students regarding self-regulation and socialization demands, particularly for those lacking a wide repertoire of SRL strategies helpful to succeed in school (Zimmerman, 2002; Cleary and Zimmerman, 2004; McClelland et al., 2006; Rosário et al., 2016). Moreover, is important to note that students from disadvantaged backgrounds (as are the students of our sample, see context and participants section) are even more vulnerable to the negative effects of the school transition from elementary to middle school (e.g., disengagement; Pendergast et al., 2018).

Supported by prior data stressing that students who self-regulate their learning are prone to be mentally active during the learning process (e.g., Rosário et al., 2010, 2017a; Azevedo et al., 2023), the current study intends to extend our knowledge on the benefits of a narrative-based intervention focused on self-regulated learning, implemented by class teachers. Teachers are suited candidates to implement educational interventions in class (Dignath et al., 2008; Skinner and Pitzer, 2012; Schuitema et al., 2016; Perry et al., 2020). Throughout the intervention, teachers are expected to help students learn SRL strategies and encourage them to use metacognitive skills; for example, helping them set goals to improve class behavior, or select the SRL strategies best suited to improve the quality of their work (Núñez et al., 2022; Tuero et al., 2022). Therefore, due to their closeness to the students’ work, teachers may play an active role in promoting student intrinsic motivation and classroom engagement (Reeve, 2012).

Taken all together, it seems relevant to train elementary school teachers to implement SRL interventions and promote students’ satisfaction of basic psychological needs and classroom engagement before their transition to middle school. Hence, the present study, following a quasi-experimental design, aims to answer the following research questions: What is the impact of the SRL intervention “Yellow’s Trials and Tribulations” (Rosário et al., 2007a,b) on (i) students’ basic psychological needs (i.e., autonomy, competence, and relatedness)?, and (ii) classroom engagement (i.e., behavioral, emotional, cognitive and agentic dimensions)? Following literature recommendations (e.g., Wang et al., 2014; Fredricks et al., 2016; Martins et al., 2021), this study (i) is grounded on the solid theoretical framework of SDT, which links students’ psychological needs and engagement (Reeve, 2012); and (ii) explores classroom engagement as a multidimensional construct by analyzing the mentioned four dimensions (Reeve and Tseng, 2011; Jang et al., 2016).

Considering the linkages between SRL and students’ engagement (e.g., Reeve and Tseng, 2011; Stefansson et al., 2018; Azevedo et al., 2023), and SDT (Reeve, 2012), it is hypothesized that the SRL intervention will benefit students’ basic psychological needs (Hypothesis 1) and classroom engagement (Hypothesis 2). Specifically, students in the experimental group are expected to report higher perceived autonomy (Hypothesis 1a), competence (Hypothesis 1b), and relatedness (Hypothesis 1c), as well as behavioral (Hypothesis 2a), emotional (Hypothesis 2b), cognitive (Hypothesis 2c) and agentic (Hypothesis 2d) classroom engagement than their counterparts in the comparison group.

Findings are expected to: (i) encourage teacher SRL training, (ii) promote the curricular infusion of SRL programs tailored to students’ educational needs, and (iii) support researchers’ and educators’ efforts to provide a classroom environment fostering learning and academically successful experiences.

## 2 Materials and methods

### 2.1 Context and participants

The current study was conducted in elementary schools in Portugal, in which the school principal applied for a national funding (Calouste Gulbenkian Foundation)^1^ for implementing evidence-based interventions in the communities. In this case, the school principal selected the narrative-based intervention “Yellow Trials and Tribulations” (Rosário et al., 2007a,b) to be implemented in 4th-grade classes. According to the available data from national statistics (PORDATA, 2018; Instituto Nacional de Estatística Censos, 2021), the participating schools are located in a region (i) with a high illiteracy rate, and low rate of a higher education degree, (ii) where individuals are likely to work on the secondary and tertiary sectors with salaries below the national average. Additionally, the school principal described the neighborhood as a “dormitory” harboring families typically showing disengagement from their children’s school life. These are relevant indicators of a disadvantaged school neighborhood (see Li and Fischer, 2017).

The assessment of the intervention in the mentioned schools followed qualitative and quantitative approaches. Cunha et al. (2023) explored the implementers’ and observers’ overall perceptions of the impact of the intervention through the qualitative analysis of the session sheets and their reflection reports about the intervention implementation. The current study examines the impact of the intervention on the participating students’ basic psychological needs and classroom engagement, analyzing quantitative data.

Ninety-six students from four 4th-grade classes participated in the intervention, however, pretest and posttest data were only available for 90 students. Hence, the experimental group is comprised of 90 students (53.3% were female, six students did not reveal this data) with ages ranging between eight and 11 years old (*M* = 9.27, SD = 0.52). The teachers of these students implemented the intervention. The teaching experience of implementers (four female teachers) ranged between 24 and 39 years (*M* = 28.25, SD = 8.26). One implementer held postgraduate training.

Following the agreement made with Gulbenkian Knowledge Academies, each applicant institution is responsible for selecting a comparison group to assess the impact of the Reference Methodology used. In this context, the coordinator of the Gulbenkian Knowledge Academy contacted the school principal of a public school district with similar sociodemographic characteristics to enroll as the comparison group. The comparison group is comprised of 91 students (52.7% were female) enrolled in six classes with ages ranging between nine and 12 years old (*M* = 9.20, SD = 0.48).

### 2.2 Procedure

The current study was approved by the Ethics Committee of the University of Minho and authorized by the Portuguese Ministry of Education. Following the Declaration of Helsinki, the guardians of the students enrolled in the experimental and comparison groups provided written informed consent to their child’s participation in the study.

Participants in both groups followed the national curriculum for the fourth-grade. The comparison group did not engage in the intervention and followed the curriculum for the fourth grade as usual. Note: the teachers of the students in this condition were not enrolled in training on SRL strategies. The teachers of the experimental group were enrolled in 50 h b-learning training between September and December 2018. The training included a theoretical part focused on motivation theories and SRL models, followed by a practical one (e.g., simulation of a session). Later, from March to June 2019, the experimental group enrolled in 10 sessions (60 min approximately) on a weekly basis, carried out in the classroom setting.

Data were collected by research assistants in the classroom context. Basic psychological needs and classroom engagement measures were collected prior to the beginning of the intervention (i.e., pretest) and at the end of the program (i.e., posttest). The implementation of the intervention was monitored by the research team through monthly videoconference sessions.

For ethical reasons, in the beginning of the following school year, the research team provided a lecture for the teachers and parents of the comparison group. The lecture was focused on the self-regulated learning processes and motivation, and was delivered through videoconference.

#### 2.2.1 “Yellow’s trials and tribulations” narrative-based intervention

The current intervention uses the story “Yellow’s Trials and Tribulations” (Rosário et al., 2007b), which narrates the adventures experienced by the colors of the rainbow while searching for their friend Yellow, who disappeared unexpectedly from the Never Ending Forest. The intervention aims to promote elementary children’s SRL strategies (e.g., goal setting, time management, and help-seeking; Rosário et al., 2017a; Cunha et al., 2021; Azevedo et al., 2022). Grounded on the social cognitive theory, the authors of the intervention advocate that students’ self-regulation and motivation are influenced by the learning environments (Rosário et al., 2007a).

Specifically, the narrative provides students with the opportunity to learn and discuss problem-solving strategies and challenges presented in contexts distinct from theirs. While discussing the story plot and the strategies used by the characters, students are encouraged to transfer the content acquired to their own learning context and life (Rosário et al., 2017a; Azevedo et al., 2022). For example, one of the chapters tells the story of a bird-teacher who encouraged bird-students to fly; “birds do not fly with closed wings,” says the bird-teacher. Through the discussion of this metaphor, which is not directly focused on the participating students’ school experiences, it is intended to elicit students’ reflection about their own behavior, and, simultaneously, instigate students’ engagement in non-academic settings and in their regular school activities (e.g., writing a composition, and solving math problems; Rosário et al., 2017a, 2019) by highlighting its relevance to learn effectively.

Throughout the narrative, some of the characters explain the processes of self-regulation, and function as role models (Bandura, 1986). For example, one of the characters of the narrative, the General-Ant, explains how the Ant Army plans, executes and evaluates their movements in the field to carry out food for their pantry in the anthill. To do all this with efficacy, the General-Ant explains that she follows the old tradition of PLEE—the theoretical model used throughout the intervention (see description below).

#### 2.2.2 The SRL model

The theoretical model used in this intervention is the PLEE (i.e., planning, execution, evaluation) cyclical model by Rosário et al. (2010). The PLEE model is based on Zimmerman’s cyclical model, which comprises three phases: forethought, performance or volitional control, and self-reflection (Zimmerman, 2002). The forethought phase requires an analysis of tasks and motivational beliefs, which means, the definition of goals, self-efficacy, and orientation toward those same goals. The performance phase, integrates self-control and self-observation skills, which translates into self-instruction, time management, and metacognitive monitoring. Finally, the self-reflection phase comprises self-judgment and self-reaction (see Zimmerman, 2002 for full description).

The three phases of the PLEE model comprise: (i) planning, in which students must think about what they plan to do, how, and when they will do it; and setting a plan for this purpose; (ii) the execution phase is displayed when the plan is put into practice; and (iii) the evaluation phase comprises the efforts to analyze the outputs against the self-set goals. Importantly, each phase of learning informs the subsequent phase, resetting the self-regulated learning cycle (Rosário et al., 2010, 2017a). This model adds a recursive nature to Zimmerman’s model. In each of the PLEE phases, individuals are expected to plan, execute, and evaluate their behaviors (e.g., during the planning phase, besides thinking and designing a plan, individuals are expected to set it, and afterward evaluate this plan of action against their self-set goals; Rosário et al., 2010, 2017a).

#### 2.2.3 Session protocol

In the current intervention, each session began with the scenario arrangement, followed by a review of the content delivered in the previous session (i.e., reviewing prior events of the story and lessons learned). Subsequently, participants were invited to read one or two chapters of the book out loud and then explore and discuss the experiences of the rainbow colors as well as the SRL processes underlying them. Finally, there was a practical activity and a take-home message. Supplementary Figure S1 provides an example of a session protocol.

The class discussions of the chapter(s) were grounded on the three types of knowledge: declarative (i.e., What is?), procedural (i.e., How?), and conditional (i.e., When? Where? Why?; Rosário et al., 2017a, 2019). This protocol allowed students to reflect on the narrative as well as on the behaviors, feelings, and accomplishments of the characters, attributing meaning and structure to their learnings while developing prospective applications of these strategies in their daily lives (Rosário et al., 2017a).

#### 2.2.4 Treatment integrity

Treatment or intervention integrity involves several procedures regarding to the adherence to protocol and implementer competence (Perepletchikova, 2011). In the current study, five procedures related to the adherence to protocol were considered: (i) intervention manual (Rosário et al., 2007a), (ii) teachers’ training, (iii) session protocol, (iv) session sheets (i.e., checklist of the session structure and white space for notes), and v) monthly practice monitoring by the research team. In the current study, it was not possible to assess the implementers’ competence during the intervention implementation.

### 2.3 Instruments and measures

#### 2.3.1 Personal data

Participants were asked about their gender and age.

#### 2.3.2 Basic psychological needs satisfaction

In order to assess each dimension of the basic psychological needs (i.e., autonomy, competence, and relatedness) items reported in previous studies were used (Reeve and Sickenius, 1994; Jang et al., 2012, 2016). Students answered this measure through a 5-point Likert scale (1 = strongly disagree, 5 = strongly agree; Jang et al., 2016). The autonomy dimension was evaluated through five items (e.g., “In this class, I feel free”; Jang et al., 2016). The competence dimension was evaluated through six items (e.g., “In this class, I feel successful in terms of completing difficult tasks”; Jang et al., 2016). Finally, the relatedness dimension was evaluated through four items (e.g., “I feel a close sense of connection with people in this class”; Jang et al., 2016). Items were originally written in English, therefore, a back translation was made to adapt the measure to the Portuguese context. Then the scale was filled out by a group of five children in order to check for comprehension. These children did not participate in the intervention study. Two items were changed to accommodate the children’s understanding. For example, the item “In this class, I feel competent” was changed to “In this class, I feel that I can do the tasks.” The scale has shown high internal consistency in previous studies (Jang et al., 2012, 2016). In the present study, the scores on this measure were also internally consistent (i.e., autonomy: α = 0.75, competence: α = 0.83, relatedness: α = 0.76).

#### 2.3.3 Classroom engagement

Classroom engagement was assessed as a multidimensional construct featuring behavioral, emotional, cognitive, and agentic dimensions. Participants answered the 19 items adapted from the engagement measure by Jang et al. (2016) using a 5-point Likert response scale (1 = strongly disagree, 5 = strongly agree). This measure focuses on students’ effort, attention, and commitment when initiating and participating in classroom learning activities as well as on their emotions throughout those activities (Jang et al., 2016). Behavioral, emotional, and agentic dimensions of engagement were assessed with five items each (e.g., “When I’m in this class, I listen very carefully.,” “When we work on something in this class, I feel interested.,” and “I let my teacher know what I need and want.,” respectively), while cognitive engagement was assessed by four items (e.g., “When reading for this class, I try to explain the key concepts in my own words.”; Jang et al., 2016). The items were also originally written in English, and a back translation was made to adapt the measure to the Portuguese context. Then the scale was filled out by a group of five children in order to check for comprehension. These children did not participate in the intervention study. Three items were changed to accommodate children’s understanding. For example, the item “I let my teacher know what I need and want” was changed to “I let my teacher know what helps me learn.” This scale has shown strong psychometric properties in a previous investigation (Jang et al., 2016). In the present study, the scores on this measure were also internally consistent (i.e., behavioral engagement: α = 0.81, emotional engagement: α = 0.82, cognitive engagement: α = 0.80, agentic engagement: α = 0.77).

### 2.4 Data analysis

The present study analyzed the impact of the intervention (i.e., independent variable) on students’ basic psychological needs (i.e., dependent variable) and classroom engagement (i.e., dependent variable). Given that the three dimensions of basic psychological needs, as well as the four dimensions of engagement, are interrelated (Ryan and Deci, 2000; Reeve, 2012), a Multivariate Analysis of Variance (MANOVA) was performed for each construct. Since data was collected at two different times (i.e., pretest and posttest) this MANOVA included repeated measures (Field, 2009). Firstly, an exploratory analysis was performed to verify the assumptions required to conduct MANOVA (Field, 2009). The statistical analyses were run using IBM SPSS version 27.0.

The effect size was calculated using the partial eta-squared coefficient (η^2^p) as described in Piñeiro et al. (2019). The coefficient values were interpreted through the Cohen (1988) benchmarks: null effect: η^2^*p* < 0.01 (*d* < 0.09); small effect: 0.01 ≤ η^2^p ≤ 0.058 (0.10 ≤ d ≤ 0.49); medium effect: 0.059 ≤ η^2^p ≤ 0.137 (0.50 ≤ d ≤ 0.79); and large effect: η^2^p ≥ 0.138 (d ≥ 0.80).

## 3 Results

Table 1 provides the descriptive statistics of all dependent variables (i.e., basic psychological needs and engagement dimensions) in the pretest and posttest for the experimental and comparison groups, respectively. Preliminary analyses were conducted to examine whether there were any differences between the two groups at the pretest. No statistically significant differences were found, which allows inferring that differences in the experimental group in the posttest can be due to the intervention.

**Table 1:** Descriptive statistics.

		Experimental group		Comparison group	
		Pretest	Posttest	Pretest	Posttest
Perceived autonomy	*M*	3.53	3.70	3.47	3.65
	SD	0.78	0.75	0.93	0.95
Perceived competence	*M*	4.08	4.42	4.17	4.20
	SD	0.60	0.71	0.69	0.76
Perceived relatedness	*M*	4.32	4.36	4.38	4.37
	SD	0.62	0.90	0.56	0.69
Behavioral engagement	*M*	4.25	4.41	4.30	4.20
	SD	0.57	0.60	0.55	0.69
Emotional engagement	*M*	4.27	4.37	4.35	4.20
	SD	0.60	0.719	0.60	0.76
Cognitive engagement	*M*	3.86	4.21	4.04	3.92
	SD	0.800	0.79	0.80	0.93
Agentic engagement	*M*	3.86	4.15	4.01	3.91
	SD	0.69	0.80	0.819	0.98

Tables 2, 3 display the correlations between the dependent variables for the experimental and comparison group, respectively. Significant Pearson correlation coefficients ranged from 0.207 to 0.798 for the experimental group, and from 0.219 to 0.831 for the comparison group. Pearson correlation coefficients between dimensions of classroom engagement are high (particularly between cognitive and agentic classroom engagement), which may indicate multicollinearity issues (see Abu-Bader, 2010). However, the results of the residuals sums-of-squares and cross-products (SSCP) matrix in MANOVA indicated that correlations are below the benchmark value of 0.80.

**Table 2:** Pearson correlation coefficients – Experimental Group (*n* = 90).

		PA		PC		PR		BE		EE		CE		AE	
	Time	1	2	1	2	1	2	1	2	1	2	1	2	1	2
1. Perceived autonomy	1	–	0.179	0.444***	0.029	0.372***	0.018	0.212*	0.119	0.312**	0.042	0.432***	0.099	0.439***	0.189
	2		–	0.184	0.600***	0.212*	0.511***	0.123	0.368***	0.207*	0.292**	0.234*	0.396***	0.253**	0.389***
2. Perceived competence	1			–	0.198	0.476***	0.126	0.626***	0.272*	0.653***	0.228*	0.592***	0.289**	0.640***	0.236*
	2				–	0.210*	0.664***	0.284**	0.565***	0.262*	0.535***	0.225*	0.571***	0.250*	0.553***
3. Perceived relatedness	1					–	0.401***	0.403***	0.318**	0.469***	0.369***	0.457***	0.356**	0.513***	0.428***
	2						–	0.210*	0.466***	0.213*	0.445***	0.141	0.444***	0.255*	0.533***
4. Behavioral engagement	1							–	0.659***	0.610***	0.389***	0.523***	0.283**	0.554***	0.320**
	2								–	0.507***	0.723***	0.464***	0.547***	0.407***	0.608***
5. Emotional engagement	1									–	0.522***	0.684***	0.372***	0.507***	0.366***
	2										–	0.396***	0.678***	0.367***	0.640***
6. Cognitive engagement	1											–	0.392***	0.727***	0.383***
	2												–	0.458***	0.798***
7. Agentic engagement	1													–	0.584***
	2														–

PA, Perceived Autonomy; PC, Perceived Competence; PR, Perceived Relatedness; BE, Behavioral Engagement; EE, Emotional Engagement; CE, Cognitive Engagement; AE, Agentic Engagement. *p < 0.05, **p < 0.01, ***p < 0.001.

**Table 3:** Pearson correlation coefficients – Comparison Group (*n* = 91).

		PA		PC		PR		BE		EE		CE		AE	
	Time	1	2	1	2	1	2	1	2	1	2	1	2	1	2
1. Perceived autonomy	1	–	0.339**	0.537***	0.323**	0.340**	0.193	0.362***	0.126	0.368***	0.173	0.341***	0.192	0.486***	0.277**
	2		–	0.385***	0.742***	0.315**	0.529***	0.383***	0.491***	0.478***	0.511***	0.448***	0.665***	0.494***	0.657***
2. Perceived competence	1			–	0.551***	0.481***	0.362***	0.630***	0.382***	0.564***	0.326**	0.665***	0.530***	0.644***	0.528***
	2				–	0.438***	0.617***	0.480***	0.636***	0.541***	0.625***	0.550***	0.783***	0.532***	0.755***
3. Perceived relatedness	1					–	0.525***	0.274**	0.219*	0.292**	0.297**	0.403***	0.333**	0.387***	0.431***
	2						–	0.305**	0.486***	0.299**	0.461***	0.399***	0.520***	0.341**	0.525***
4. Behavioral engagement	1							–	0.558***	0.683***	0.387***	0.641***	0.441***	0.554***	0.387***
	2								–	0.455***	0.764***	0.448***	0.544***	0.390***	0.494***
5. Emotional engagement	1									–	0.523***	0.570***	0.489***	0.568***	0.510***
	2										–	0.423***	0.633***	0.455***	0.573***
6. Cognitive engagement	1											–	0.637***	0.729***	0.634***
	2												–	0.608***	0.831***
7. Agentic engagement	1													–	0.696***
	2														–

PA, Perceived Autonomy; PC, Perceived Competence; PR, Perceived Relatedness; BE, Behavioral Engagement; EE, Emotional Engagement; CE, Cognitive Engagement; AE, Agentic Engagement. *p < 0.05, **p < 0.01, ***p < 0.001.

Regarding basic psychological needs, results indicate no statistically significant multivariate group effect (Table 4), Wilks’ Lambda = 0.991, *F*(3, 175) = 0.536, *p* = 0.658, η^2^p = 0.009; moreover, a statistically significant multivariate time effect, Wilks’ Lambda = 0.926, *F* (3, 175) = 4.677, *p* = 0.004, η^2^p = 0.074, and a statistically significant multivariate group × time interaction effect were found, Wilks’ Lambda = 0.942, *F*(3, 175) = 3.578, *p* = 0.015, η^2^p = 0.058.

**Table 4:** Summary of basic psychological needs univariate analyses of repeated measures.

	Group effect		Time effect		Time × Group effect	
	*F*	*p*	*F*	*p*	*F*	*p*
Perceived autonomy	0.23	0.63	5.28	<0.05	0.00	0.95
Perceived competence	0.55	0.46	10.41	<0.001	6.99	<0.01
Perceived relatedness	0.14	0.70	0.08	0.78	0.18	0.67

**Figure 1:**
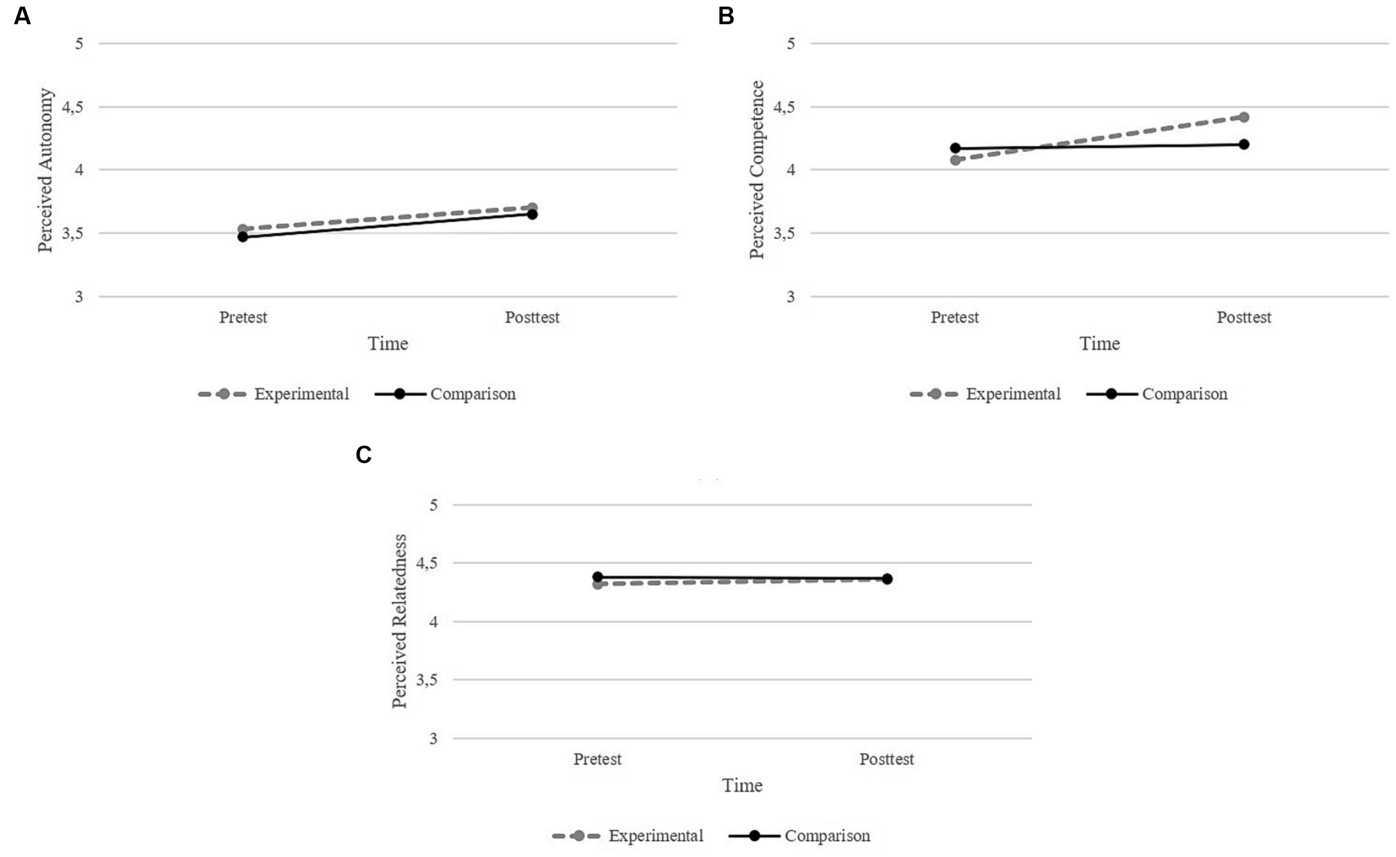
Graphical representation of Perceived Autonomy **(A)**, Perceived Competence **(B),** and Perceived Relatedness **(C)** over time (pretest-posttest).

Univariate results revealed that of the three basic psychological needs, perceived autonomy and perceived competence had statistically significant results, while perceived relatedness had no statistically significant effects (see Figures 1A–C). A significant effect of time on perceived autonomy was found, *F*(1, 177) = 5.81, *p* < 0.05, η^2^p = 0.029. Data also showed a significant effect of time in perceived competence, *F*(1, 177) = 10.405, *p* < 0.001, η^2^p = 0.056, and of group x time interaction in perceived competence, *F*(1, 177) = 6.994, *p* < 0.01, η^2^p = 0.038. Regarding these two variables, pairwise comparisons revealed that both groups increased perceived autonomy over time, however in the posttest the groups did not differ (see Figure 1A). Pairwise comparisons also revealed that from pretest to posttest, students in the experimental group reported higher perceived competence than students in the comparison group (see Figure 1B).

Concerning engagement, no statistically significant multivariate group effect was found (Table 5), Wilks’ Lambda = 0.993, *F*(4, 174) = 0.295, *p* = 0.881, η^2^p = 0.007; moreover, no statistically significant multivariate time effect was found, Wilks’ Lambda = 0.955, *F*(4, 174) = 2.032, *p* = 0.092, η^2^p = 0.045, and a statistically significant multivariate group x time interaction effect were found, Wilks’ Lambda = 0.899, *F*(4, 174) = 4.898, *p* = 0.001, η^2^p = 0.101.

**Table 5:** Summary of engagement univariate analyses of repeated measures.

	Group effect		Time effect		Time × Group effect	
	*F*	*p*	*F*	*p*	*F*	*p*
Behavioral engagement	1.10	0.30	0.62	0.43	9.74	<0.01
Emotional engagement	0.31	0.58	0.21	0.65	6.11	<0.05
Cognitive engagement	0.24	0.63	3.99	<0.05	14.51	<0.001
Agentic engagement	0.20	0.66	3.20	0.08	13.59	<0.001

**Figure 2:**
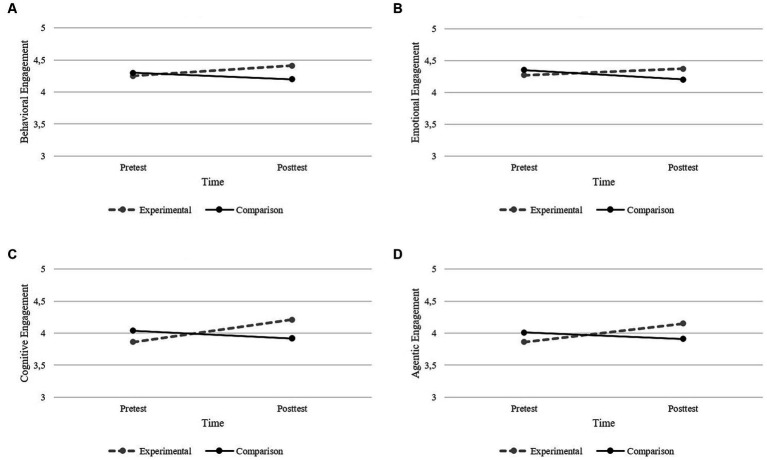
Graphical representation of the levels of Behavioral Engagement **(A)**, Emotional Engagement **(B)**, Cognitive Engagement **(C)**, and Agentic Engagement **(D)** over time (from pretest to posttest).

Univariate results showed a significant effect of group x time interaction in behavioral engagement, *F*(1, 177) = 9.743, *p* < 0.01, η^2^p = 0.052, emotional engagement, *F*(1, 177) = 6.111, *p* < 0.05, η^2^p = 0.033, and agentic engagement, *F*(1, 177) = 13.589, *p* < 0.001, η^2^p = 0.071. Data also reported a significant effect of time, *F*(1, 177) = 3.985, *p* < 0.05, η^2^p = 0.022, and group × time interaction, *F*(1, 177) = 14.514, *p* < 0.001, η^2^p = 0.076, in cognitive engagement. Regarding these engagement variables, pairwise comparisons showed an increase in the experimental group, from pretest to posttest, in the reported behavioral, emotional, cognitive, and agentic engagement (see Figures 2A–D). The comparison group revealed a statistically significant decrease in the reported emotional engagement from pretest to posttest (see Figure 1B).

## 4 Discussion

The current study aimed to assess the impact of the narrative-based intervention “Yellow’s Trials and Tribulations,” implemented by fourth-grade class teachers, on their students’ basic psychological needs satisfaction and classroom engagement. Grounded on prior literature (Fitzpatrick, 2012; Rosário et al., 2016; Azevedo et al., 2023), we hypothesized that students who benefited from SRL training would report higher basic psychological needs satisfaction (Hypothesis 1) and classroom engagement (Hypothesis 2) than their counterparts in the comparison group.

Regarding basic psychological needs, the study hypotheses were partially confirmed. No statistically significant differences were found between the experimental and comparison groups in two variables of the students’ basic psychological needs at the end of the intervention: perceived autonomy and relatedness. At first glance, these results are surprising given the purpose and protocol of the intervention, and the qualitative findings gathered from the implementers’ and observers’ notes of the intervention implementation. The current intervention provides several opportunities for students to share their thoughts and opinions while learning SRL strategies. For this reason, the intervention was expected to help students experience choice in their actions, enthusiasm, and appreciation (Skinner and Belmont, 1993; Ryan and Deci, 2020). Moreover, qualitative findings indicated that implementers and observers reported several examples of students who participated by sharing their opinions during intervention sessions and in class (Cunha et al., 2023). However, students’ level of perceived autonomy during the intervention and the remaining instruction time (i.e., regular classes) may be different. Possibly, teachers’ motivational style during instruction time may not facilitate students’ perceived autonomy (e.g., Jang et al., 2016) as much as during intervention time. As a result, students may not have perceived as much autonomy in their class when they completed the questionnaire (e.g., “In this class, I feel free”; Jang et al., 2016). Possibly for this reason, students from the experimental and comparison groups did not differ in the posttest. Regarding perceived relatedness, although qualitative findings revealed an enhancement of students’ peer relationships, data was not focused on the relationship with their class teacher - the intervention implementer (Cunha et al., 2023). This finding may explain the lack of statistical significance in the current study, given that no distinction was made between classmates and teacher relationship in the questionnaire used (e.g., “I feel a close sense of connection with people in this class”; Jang et al., 2016).

However, statistically significant differences were found for perceived competence (Hypothesis 1b). At the end of the intervention, students enrolled in the experimental group reported higher perceived competence than the students from the comparison group (although with a small effect size). This positive result is consistent with the qualitative findings that indicated that students who participated in the intervention were perceived by the implementers (i.e., teachers) and observers as being more confident and participating more in class, even for students with low prior achievement (Cunha et al., 2023). Students’ acquisition of SRL strategies may have empowered them to feel more confident in their competence to participate, and the positive feedback from the implementer during the session discussions may have contributed to satisfying their need for competence (Skinner and Belmont, 1993; Ryan and Deci, 2020). Practical activities were also planned to provide diverse and optimally challenging opportunities for students to apply the SRL strategies trained in the session. This protocol may also have contributed to increasing students’ perceived competence (Skinner and Belmont, 1993; Cook and Artino, 2016; Ryan and Deci, 2020).

Following the proposition that basic psychological needs are an antecedent of engagement (Deci and Ryan, 2000; Reeve, 2012; Ryan and Deci, 2020), it is possible to conclude that when basic psychological needs are satisfied, students are more likely to engage in the classroom learning activities (Deci and Ryan, 2000; Reeve, 2012; Reeve and Lee, 2014). Despite not conducting a mediation analysis (e.g., Jang et al., 2012), the improvement found in perceived competence may have contributed to students’ classroom engagement. Regarding this construct, statistically significant results were found for all engagement dimensions (i.e., Hypotheses 2a-d), which is especially relevant considering that students are from a disadvantaged school neighborhood. The quantitative results retrieved from all participating students substantiate prior anecdotical qualitative findings of the implementers’ and observers’ overall perceived impact of the intervention on students (Cunha et al., 2023). For instance, qualitative data (Cunha et al., 2023) provided some evidence of students’ participation in session and class discussions (i.e., behavioral classroom engagement), positive emotions regarding progresses and learning (i.e., emotional classroom engagement), application of self-regulation strategies during their study (i.e., cognitive engagement), and a growing willingness to share their thoughts and opinions (i.e., agentic engagement).

Notwithstanding the current positive impact of the intervention, the effect sizes found were small (i.e., behavioral and emotional engagement) and medium (i.e., cognitive and agentic engagement), depending on the engagement dimensions analyzed. These results contrast with prior research showing large effect sizes of the intervention on students’ school engagement (Rosário et al., 2016; Azevedo et al., 2023). Those results could be related to distinct reasons (e.g., different outcome measures, methods of data collection, and implementers of the intervention). Regarding outcome measures and data collection methods, prior studies were focused on general school engagement instead of a restricted level of engagement. For example, in the study by Rosário et al. (2016), behavioral engagement was measured through classroom observations (several times throughout the school year) that focused on students’ class attendance and punctuality, body language evidencing attention, and compliance with the class routines and rules; while in the study by Azevedo et al. (2023), behavioral engagement was measured using students’ self-report of the level of distraction in schoolwork, and school records of students’ class attendance and punctuality. In the case of the current study, behavioral engagement was measured through students’ self-reports which focused on attention, effort, and participation in class. Moreover, according to the literature, the implementer (researcher vs. class teacher) could also be a major factor in helping explain the different effect sizes found. Contrary to prior works where researchers acted as implementors of the intervention (Rosário et al., 2016; Azevedo et al., 2023), in the current study, class teachers were the implementers. According to extant meta-analyses (see Dignath and Büttner, 2008; de Boer et al., 2018), particularly those conducted at elementary school level, interventions conducted by researchers rather than by class teachers are more effective regarding students’ overall academic performance, reading or writing performance and strategy use (Dignath et al., 2008). At the same time, the intervention implemented by teachers has distinct strengths (e.g., teachers can keep encouraging students to use metacognitive skills during their work in class) as previously mentioned (Núñez et al., 2022; Tuero et al., 2022). In the school context, it is important to monitor and assess the impact of the intervention and identify aspects that need improvement. Current results provide some concrete implications for practice as described below.

### 4.1 Strengths, limitations, and implications

The current study, due to its interventional nature, adds to SRL and engagement literature, extending our knowledge on the impact of a SRL narrative-based intervention on students’ basic psychological needs and four dimensions of classroom engagement. Moreover, this work added the agentic engagement dimension which helps highlight the contribution of the intervention in promoting students’ intentional, proactive, and constructive actions in the classroom environment (Reeve and Tseng, 2011). This is consistent with the social cognitive theoretical framework of the intervention in which students are the authors of their learning path (Bandura, 1986; Rosário et al., 2010, 2017a). This sense of agency is essential to overcome challenges typically experienced by students from disadvantaged backgrounds such as those of the current participating students.

Despite the strengths of the current study, some limitations as well as implications for future research and practice should be addressed. The first limitation is related to data collection. Students reported their basic psychological needs satisfaction and classroom engagement in two moments (pre-and post-intervention), but follow-up data were not collected. Therefore, while the intervention led to positive results, future studies could consider investigating its long-term effects by planning quasi-experimental designs with follow-up measures (at least 3 months after the intervention, Tuero et al., 2022).

Moreover, no data addressing intervention-focused students’ psychological needs satisfaction and engagement were collected. The self-reported measures collected (i.e., basic psychological needs and classroom engagement) were focused on the classroom context and they do not capture the specificities of students’ psychological needs and engagement in the SRL intervention. Therefore, future studies may consider using self-report measures focused on the intervention to capture students’ psychological needs and engagement processes (e.g., students’ participation during session discussions, peer relationships) during the intervention sessions (see Cunha et al., 2023). This would allow analyzing differences in these two variables, as students may perceive their psychological needs satisfaction and engagement differently according to the context (i.e., class vs. intervention). Collecting these data could be of particular relevance when the intervention implementers are teachers (as in the current study), because it can be used to extend their work by transferring the knowledge and intentionality applied in the intervention into the classroom context. This strategy is expected to contribute to maximizing the positive impact of the intervention (e.g., Dignath et al., 2008).

Additionally, in the current study, the implementers’ competence was not assessed as recommended by Perepletchikova (2011). According to this work, the assessment of implementers’ competence is an essential procedure to ensure treatment integrity by contributing to the avoidance of ambiguous interpretations regarding the evidenced-based practices implemented and intervention effectiveness. The implementers’ competence to deliver interventions following the protocols (to achieve the pre-established goals) is of high importance to the intervention’s effects. Grounded on this knowledge, future intervention studies may consider including direct (e.g., through observations, videotaping) or indirect assessment methods (e.g., checklists) to assess implementers’ (e.g., researchers, teachers or other educators) competence in effectively implementing the intervention. These assessment methods could be used at different moments of the intervention (i.e., before, during, and after the end of the intervention) functioning as a tool for researchers and implementers. They could evaluate the adherence to the session protocol (i.e., implementation of specific procedures, tasks, and activities), monitor competences in delivering the intervention sessions (e.g., flexibility to administrate some tasks), and consequently adjust practices if needed. For instance, implementer-teachers could consider using checklists to self-monitor the competences needed to implement the intervention efficaciously. In the case of the current intervention, examples of checklist statements addressing the three basic psychological needs (Deci and Ryan, 2000; Ryan and Deci, 2020) could be: during the session (i) “I provided students the opportunity to choose their character when reading the book chapter;” (autonomy), (ii) “I provided students with positive and constructive feedback” (competence), and (iii) “I welcomed students answers and respected different opinions” (relatedness). By checking this type of statements, implementer-teachers are expected to reflect upon their approaches to students during each session and make the necessary adjustments to improve their performance on the promotion of psychological needs satisfaction. Note that implementation and integrity procedures, in particular the use of checklists to evaluate implementers’ competence, should be carefully explained to the implementer-teachers before the beginning of the intervention implementation. This should be done in order to ensure that implementers perceive checklists as a work tool to improve their competence to deliver the intervention and not a mechanism for researchers to exert control over their sessions (Cunha et al., 2023). In sum, data gathered from checklists could have helped to further understand the results found, particularly, those non-statistically significant (e.g., students’ perceived autonomy and relatedness).

Finally, implementers need time and opportunities to practice, consolidate, adjust their practice, and progressively increase their self-efficacy to implement effectively the intervention. For this reason, school administrators need to understand the implementation of school-based interventions as an investment in the long-term, managing resources and training opportunities to the benefit of students.

## Data availability statement

The datasets presented in this article are not readily available because informed consent stated that only the research team would have access to the data. Requests to access the datasets should be directed to PR, prosario@psi.uminho.pt.

## Ethics statement

The studies involving humans were approved by Ethics Committee of the University of Minho. The studies were conducted in accordance with the local legislation and institutional requirements. Written informed consent for participation in this study was provided by the participants’ legal guardians/next of kin.

## Author contributions

JC conceived the idea and design of the study. JC, JM, and PR were responsible for teachers’ training, and practice monitoring. JC, JM, and RP were responsible for the literature search, collection, analysis, and interpretation of data for the work. JC, JM, and RP wrote the manuscript. PR was in charge of technical guidance and made an important intellectual contribution to manuscript revision. All authors contributed to the article and approved the submitted version.

## Footnotes

^1^The mission of the CGF is to work for a fairer and more sustainable society, improving people’s quality of life through art, charity, science, and education. The Foundation develops a vast activity through its own projects or in partnership with other entities providing grants and scholarships to institutions and social organizations. The intervention strategy between 2018 and 2022 comprised three priority areas: social cohesion and integration, sustainability, and knowledge (Calouste Gulbenkian Foundation, 2019). Integrated into the 2018–2022 intervention strategy, in 2018, a new project called GKA emerged. The purpose was to empower children and youth (up to 25 years old) by promoting social and emotional skills not covered in the regular school curriculum, such as adaptability, communication, creative thinking, resilience, problem-solving, and self-regulation (Calouste Gulbenkian Foundation, 2019).

## References

[ref1] Abu-Bader S. H. (2010). Advanced and multivariate statistical methods for social science research (New York: Oxford University Press).

[ref2] Appleton J. J. Christenson S. L. Kim D. Reschly A. L. (2006). Measuring cognitive and psychological engagement: validation of the student engagement instrument. J. Sch. Psychol. 44, 427–445. doi: 10.1016/j.jsp.2006.04.002

[ref3] Archambault I. Dupéré V. (2017). Joint trajectories of behavioral, affective, and cognitive engagement in elementary school. J. Educ. Res. 110, 188–198. doi: 10.1080/00220671.2015.1060931

[ref4] Azevedo R. Rosário P. Magalhães P. Núñez J. C. Pereira B. Pereira A. (2022). A tool-kit to help students from low socioeconomic status background: a school-based self-regulated learning intervention. Eur. J. Psychol. Educ. 38, 495–518. doi: 10.1007/s10212-022-00607-y

[ref5] Azevedo R. Rosário P. Núñez J. C. Vallejo G. Fuentes S. Magalhães P. (2023). A school-based intervention on elementary students’ school engagement. Contemp. Educ. Psychol. 73:102148. doi: 10.1016/j.cedpsych.2023.102148

[ref6] Bandura A. (1986). The explanatory and predictive scope of self-efficacy theory. J. Soc. Clin. Psychol. 4, 359–373. doi: 10.1521/jscp.1986.4.3.359

[ref7] Ben-Eliyahu A. Moore D. Dorph R. Schunn C. D. (2018). Investigating the multidimensionality of engagement: affective, behavioral, and cognitive engagement across science activities and contexts. Contemp. Educ. Psychol. 53, 87–105. doi: 10.1016/j.cedpsych.2018.01.002

[ref8] Calouste Gulbenkian Foundation (2019). Gulbenkian academies for knowledge. Available at: https://cdn.gulbenkian.pt/academias/wp-content/uploads/sites/43/2019/07/ACG_BrochuraEN.pdf (Accessed April 20, 2023)

[ref9] Cleary T. J. Zimmerman B. J. (2004). Self-regulation empowerment program: a school-based program to enhance self-regulated and self-motivated cycles of student learning. Psychol. Sch. 41, 537–550. doi: 10.1002/pits.10177

[ref10] Cleary T. J. Zimmerman B. J. (2012). “A cyclical self-regulatory account of student engagement: theoretical foundations and applications” in Handbook of research on student engagement. eds. Christenson S. L. Reschly A. L. Wylie C. (Boston: Springer US), 237–257.

[ref11] Cohen J. (1988). Statistical power analysis for the behavioral sciences. (Hillsdale, NJ: Routledge).

[ref12] Conesa P. J. Duñabeitia J. A. (2021). The basic psychological needs in the classroom scale (BPN-CS). Behav. Sci. 11:96. doi: 10.3390/bs11070096, PMID: 34202640 PMC8301179

[ref13] Cook D. Artino A. (2016). Motivation to learn: an overview of contemporary theories. Med. Educ. 50, 997–1014. doi: 10.1111/medu.13074, PMID: 27628718 PMC5113774

[ref14] Côté-Lussier C. Fitzpatrick C. (2016). Feelings of safety at school, socioemotional functioning, and classroom engagement. J. Adolesc. Health 58, 543–550. doi: 10.1016/j.jadohealth.2016.01.003, PMID: 26976149

[ref15] Cristóvão A. M. Candeias A. A. Verdasca J. (2017). Social and emotional learning and academic achievement in Portuguese schools: a bibliometric study. Front. Psychol. 8:1913. doi: 10.3389/fpsyg.2017.01913, PMID: 29167650 PMC5682338

[ref16] Cunha J. Guimarães A. Martins J. Rosário P. (2023) A self-regulation intervention conducted by teachers in a disadvantaged school neighborhood: implementers’ and observers’ perceptions of its impact on elementary students. Children, 10:1795. doi: 10.3390/children10111795 38002886 PMC10670183

[ref17] Cunha J. Silva C. Guimarães A. Sousa P. Vieira C. Lopes D.. (2021). No children should be left behind during COVID-19 pandemic: description, potential reach, and participants’ perspectives of a project through radio and letters to promote self-regulatory competences in elementary school. Front. Psychol. 12, 647–708. doi: 10.3389/fpsyg.2021.647708, PMID: 34025518 PMC8131506

[ref18] de Boer H. Donker A. S. Kostons D. D. N. M. D. N. M. van der Werf G. P. C. (2018). Long-term effects of metacognitive strategy instruction on student academic performance: a meta-analysis. Educ. Res. Rev. 24, 98–115. doi: 10.1016/j.edurev.2018.03.002

[ref19] Deci E. L. Ryan R. M. (2000). The “what” and “why” of goal pursuits: human needs and the self-determination of behavior. Psychol. Inq. 11, 227–268. doi: 10.1207/S15327965PLI1104_01

[ref20] Dignath C. Buettner G. Langfeldt H.-P. (2008). How can primary school students learn self-regulated learning strategies most effectively?: a meta-analysis on self-regulation training programmes. Educ. Res. Rev. 3, 101–129. doi: 10.1016/j.edurev.2008.02.003

[ref21] Dignath C. Büttner G. (2008). Components of fostering self-regulated learning among students. A meta-analysis on intervention studies at primary and secondary school level. Metacogn. Learn. 3, 231–264. doi: 10.1007/s11409-008-9029-x

[ref22] Estévez I. Rodríguez-Llorente C. Piñeiro I. González-Suárez R. Valle A. (2021). School engagement, academic achievement, and self-regulated learning. Sustainability 13:3011. doi: 10.3390/su13063011

[ref23] Field A. P. (2009). Discovering statistics using SPSS. London: SAGE.

[ref24] Fitzpatrick C. (2012). Ready or not: kindergarten classroom engagement as an indicator of child school readiness. S. Afr. J. Child. Educ. 2, 1–32. doi: 10.4102/sajce.v2i1.19

[ref25] Fredricks J. A. Blumenfeld P. C. Friedel J. Paris A. H. (2005). “School engagement” in What do children need to flourish: Conceptualizing and measuring indicators of positive development. eds. Moore K. A. Lippman L. H. (Springer Science), 305–321.

[ref26] Fredricks J. A. Blumenfeld P. C. Paris A. H. (2004). School engagement: potential of the concept, state of the evidence. Rev. Educ. Res. 74, 59–109. doi: 10.3102/00346543074001059

[ref27] Fredricks J. A. Filsecker M. Lawson M. A. (2016). Student engagement, context, and adjustment: addressing definitional, measurement, and methodological issues. Learn. Instr. 43, 1–4. doi: 10.1016/j.learninstruc.2016.02.002

[ref28] Fredricks J. A. McColskey W. (2012). “The measurement of student engagement: a comparative analysis of various methods and student self-report instruments” in Handbook of research on student engagement. eds. Christenson S. L. Reschly A. L. Wylie C. (New York: Springer), 763–782.

[ref29] Fredricks J. A. Reschly A. L. Christenson S. L. (2019). “Interventions for student engagement: overview and state of the field” in Handbook of student engagement interventions: working with disengaged students. (London: Academic Press), 1–11.

[ref31] Hill C. J. Bloom H. S. Black A. R. Lipsey M. W. (2008). Empirical benchmarks for interpreting effect sizes in research. Child Dev. Perspect. 2, 172–177. doi: 10.1111/j.1750-8606.2008.00061.x

[ref9001] Hughes J. N. Luo W. Kwok O. M. Loyd L. K. (2008). Teacher-student support, effortful engagement, and achievement: a 3-year longitudinal study. J Educ Psychol. 100, 1–14. doi: 10.1037/0022-0663.100.1.1 19578558 PMC2705122

[ref33] Hulleman C. S. Barron K. E. (2016). “Motivation interventions in education: bridging theory, research, and practice” in Handbook of educational psychology. eds. Corno L. Anderman E. M. (New York: Routledge), 160–171.

[ref34] Instituto Nacional de Estatística Censos (2021). Available at: https://tabulador.ine.pt/censos2021/ (Accessed April 21, 2023).

[ref35] Jang H. Kim E. J. Reeve J. (2012). Longitudinal test of self-determination theory’s motivation mediation model in a naturally occurring classroom context. J. Educ. Psychol. 104, 1175–1188. doi: 10.1037/a0028089

[ref36] Jang H. Kim E. J. Reeve J. (2016). Why students become more engaged or more disengaged during the semester: a self-determination theory dual-process model. Learn. Instr. 43, 27–38. doi: 10.1016/j.learninstruc.2016.01.002

[ref38] Lazowski R. A. Hulleman C. S. (2015). Motivation interventions in education: a meta-analytic review. Rev. Educ. Res. 86, 602–640. doi: 10.3102/0034654315617832

[ref39] Li A. Fischer M. J. (2017). Advantaged/disadvantaged school neighborhoods, parental networks, and parental involvement at elementary school. Sociol. Educ. 90, 355–377. doi: 10.1177/0038040717732332

[ref40] Luo W. Hughes J. N. Liew J. Kwok O. (2009). Classifying academically at-risk first graders into engagement types: association with long-term achievement trajectories. Elem. Sch. J. 109, 380–405. doi: 10.1086/593939, PMID: 19343104 PMC2663942

[ref41] Marks H. M. (2000). Student engagement in instructional activity: patterns in the elementary, middle, and high school years. Am. Educ. Res. J. 37, 153–184. doi: 10.3102/00028312037001153

[ref42] Martins J. Cunha J. Lopes S. Moreira T. Rosário P. (2021). School engagement in elementary school: a systematic review of 35 years of research. Educ. Psychol. Rev. 34, 793–849. doi: 10.1007/s10648-021-09642-5

[ref43] Martins J. Rosário P. Cunha J. Núñez J. C. Vallejo G. Moreira T. (2023). How to help students in their transition to middle school? Effectiveness of a school-based group mentoring program promoting students’ engagement, self-regulation, and goal setting. Contemp. Educ. Psychol. 102230. doi: 10.1016/j.cedpsych.2023.102230

[ref44] McClelland M. M. Acock A. C. Morrison F. J. (2006). The impact of kindergarten learning-related skills on academic trajectories at the end of elementary school. Early Child Res. Q. 21, 471–490. doi: 10.1016/j.ecresq.2006.09.003

[ref45] Mullender-Wijnsma M. J. Hartman E. de Greeff J. W. Bosker R. J. Doolaard S. Visscher C. (2015). Moderate-to-vigorous physically active academic lessons and academic engagement in children with and without a social disadvantage: a within subject experimental design. BMC Public Health 15, 1–9. doi: 10.1186/s12889-015-1745-y 25927371 PMC4412042

[ref46] Niemiec C. P. Ryan R. M. (2009). Autonomy, competence, and relatedness in the classroom: applying self-determination theory to educational practice. Theory Res. Edu. 7, 133–144. doi: 10.1177/1477878509104318

[ref47] Núñez J. C. Tuero E. Fernández E. Añón F. J. Manalo E. Rosário P. (2022). Effect of an intervention in self-regulation strategies on academic achievement in elementary school: a study of the mediating effect of self-regulatory activity. Rev. Psicodidáct. Engl. Ed 27, 9–20. doi: 10.1016/j.psicoe.2021.09.001

[ref48] Pagani L. Fitzpatrick C. Archambault I. Janosz M. (2010). School readiness and later achievement: a French Canadian replication and extension. Dev. Psychol. 46, 984–994. doi: 10.1037/a0018881, PMID: 20822217

[ref49] Pekrun R. (2020). Commentary: self-report is indispensable to assess students’ learning. Frontline Learn. Res. 8, 185–193. doi: 10.14786/flr.v8i3.637

[ref50] Pendergast D. Allen J. McGregor G. Ronksley-Pavia M. (2018). Engaging marginalized, “at-risk” middle-level students: a focus on the importance of a sense of belonging at school. Educ. Sci. 8:138. doi: 10.3390/educsci8030138

[ref51] Pereira A. Miranda S. Teixeira S. Mesquita S. Zanatta C. Rosário P. (2021). Promote selective attention in 4th-grade students: lessons learned from a school-based intervention on self-regulation. Children 8:182. doi: 10.3390/children8030182, PMID: 33804331 PMC8000716

[ref52] Perepletchikova F. (2011). On the topic of treatment integrity. Clin. Psychol. Publ. Div. Clin. Psychol. Am. Psychol. Assoc. 18, 148–153. doi: 10.1111/j.1468-2850.2011.01246.x, PMID: 21769167 PMC3137485

[ref53] Perry N. E. Lisaingo S. Yee N. Parent N. Wan X. Muis K. (2020). Collaborating with teachers to design and implement assessments for self-regulated learning in the context of authentic classroom writing tasks. Assessment Educ. Principles Policy Pract. 27, 416–443. doi: 10.1080/0969594X.2020.1801576

[ref54] Piñeiro I. Estévez I. Freire C. de Caso A. Souto A. González-Sanmamed M. (2019). The role of prior achievement as an antecedent to student homework engagement. Front. Psychol. 10:140. doi: 10.3389/fpsyg.2019.00140, PMID: 30774613 PMC6367270

[ref55] Pino-James N. Shernoff D. J. Bressler D. M. Larson S. C. Sinha S. (2019). “Chapter 8 - instructional interventions that support student engagement: an international perspective” in Handbook of student engagement interventions. eds. Fredricks J. A. Reschly A. L. Christenson S. L. (London: Academic Press), 103–119.

[ref56] PORDATA (2018). Retrato de Portugal PORDATA. Fundação Manuel dos Santos. Available at: https://www.pordata.pt/Retratos/2018/Retrato+de+Portugal-74 (Accessed April, 22 2023).

[ref58] Reeve J. Lee W. (2014). Students’ classroom engagement produces longitudinal changes in classroom motivation. J. Educ. Psychol. 106, 527–540. doi: 10.1037/a0034934

[ref59] Reeve J. Sickenius B. (1994). Development and validation of a brief measure of the three psychological needs underlying intrinsic motivation: the Afs scales. Educ. Psychol. Meas. 54, 506–515. doi: 10.1177/0013164494054002025

[ref57] Reeve J. (2012). “A self-determination theory perspective on student engagement” in Handbook of research on student engagement. eds. Christenson S. L. Reschly A. L. Wylie C. (New York: Springer US), 149–172.

[ref60] Reeve J. Tseng C.-M. (2011). Agency as a fourth aspect of students’ engagement during learning activities. Contemp. Educ. Psychol. 36, 257–267. doi: 10.1016/j.cedpsych.2011.05.002

[ref61] Reyna V. F. Brainerd C. J. (2007). The importance of mathematics in health and human judgment: numeracy, risk communication, and medical decision making. Learn. Individ. Differ. 17, 147–159. doi: 10.1016/j.lindif.2007.03.010

[ref62] Rosário P. Högemann J. Núñez J. C. Vallejo G. Cunha J. Rodríguez C.. (2019). The impact of three types of writing intervention on students’ writing quality. PLoS One 14:e0218099. doi: 10.1371/journal.pone.0218099, PMID: 31318868 PMC6638999

[ref63] Rosário P. Núñez J. C. González-Pienda J. A. (2007a). Auto-regulação em crianças sub-10: Projecto Sarilhos do Amarelo. Porto: Porto Editora.

[ref64] Rosário P. Núñez J. C. González-Pienda J. A. (2007b). Sarilhos do Amarelo [Yellow’s trials and tribulations]. Porto: Porto Editora.

[ref65] Rosário P. Núñez J. C. González-Pienda J. A. Valle A. (2010). “Enhancing primary school students self-regulated learning: Yellow’s trials and tribulations project” in International perspectives on applying self-regulated learning in different settings (Almeria: Education and Psychology), 139–156.

[ref66] Rosário P. Núñez J. C. Rodríguez C. Cerezo R. Fernández E. Tuero E.. (2017a). Analysis of instructional programs in different academic levels for improving self-regulated learning SRL through written text. Brill. 201–231. doi: 10.1163/9789004270480_010

[ref67] Rosário P. Núñez J. C. Vallejo G. Azevedo R. Pereira R. Moreira T.. (2017b). Promoting gypsy children’s behavioural engagement and school success: evidence from a four-wave longitudinal study. Br. Educ. Res. J. 43, 554–571. doi: 10.1002/berj.3271

[ref68] Rosário P. Núñez J. C. Vallejo G. Cunha J. Azevedo R. Pereira R.. (2016). Promoting gypsy children school engagement: a story-tool project to enhance self-regulated learning. Contemp. Educ. Psychol. 47, 84–94. doi: 10.1016/j.cedpsych.2015.11.005

[ref69] Ryan R. M. Deci E. L. (2000). Self-determination theory and the facilitation of intrinsic motivation, social development, and well-being. Am. Psychol. 55, 68–78. doi: 10.1037/0003-066X.55.1.68, PMID: 11392867

[ref70] Ryan R. M. Deci E. L. (2020). Intrinsic and extrinsic motivation from a self-determination theory perspective: definitions, theory, practices, and future directions. Contemp. Educ. Psychol. 61:101860. doi: 10.1016/j.cedpsych.2020.101860

[ref71] Sala A. Punie Y. Garkov V. Cabrera M. (2020). LifeComp: The European Framework for Personal, Social and Learning to Learn Key Competence, *EUR 30246 EN*, (Luxembourg: Publications Office of the European Union). doi: 10.2760/922681

[ref72] Santos A. C. Simões C. Cefai C. Freitas E. Arriaga P. (2021). Emotion regulation and student engagement: age and gender differences during adolescence. Int. J. Educ. Res. 109:101830. doi: 10.1016/j.ijer.2021.101830

[ref73] Schuitema J. Peetsma T. van der Veen I. (2016). Longitudinal relations between perceived autonomy and social support from teachers and students’ self-regulated learning and achievement. Learn. Individ. Differ. 49, 32–45. doi: 10.1016/j.lindif.2016.05.006

[ref74] Skinner E. A. Belmont M. J. (1993). Motivation in the classroom: reciprocal effects of teacher behavior and student engagement across the school year. J. Educ. Psychol. 85, 571–581. doi: 10.1037/0022-0663.85.4.571

[ref75] Skinner E. A. Kindermann T. A. Furrer C. J. (2009). A motivational perspective on engagement and disaffection: conceptualization and assessment of children’s behavioral and emotional participation in academic activities in the classroom. Educ. Psychol. Meas. 69, 493–525. doi: 10.1177/0013164408323233

[ref76] Skinner E. A. Pitzer J. R. (2012). “Developmental dynamics of student engagement, coping, and everyday resilience” in Handbook of research on student engagement. eds. Christenson S. L. Reschly A. L. Wylie C. (Boston: Springer US), 21–44.

[ref77] Skinner E. A. Pitzer J. R. Steele J. S. (2016). Can student engagement serve as a motivational resource for academic coping, persistence, and learning during late elementary and early middle school? Dev. Psychol. 52, 2099–2117. doi: 10.1037/dev0000232, PMID: 27893248

[ref78] Stefansson K. K. Gestsdottir S. Birgisdottir F. Lerner R. M. (2018). School engagement and intentional self-regulation: a reciprocal relation in adolescence. J. Adolesc. 64, 23–33. doi: 10.1016/j.adolescence.2018.01.005, PMID: 29408096

[ref79] Tuero E. Núñez J. C. Vallejo G. Fernández M. P. Añón F. J. Moreira T.. (2022). Short and long-term effects on academic performance of a school-based training in self-regulation learning: a three-level experimental study. Front. Psychol. 13:889201. doi: 10.3389/fpsyg.2022.889201, PMID: 35645884 PMC9134005

[ref80] Van den Broeck A. Ferris D. L. Chang C.-H. Rosen C. C. (2016). A review of self-determination theory’s basic psychological needs at work. J. Manage. 42, 1195–1229. doi: 10.1177/0149206316632058

[ref82] Wang M.-T. Hofkens T. L. (2020). Beyond classroom academics: a school-wide and multi-contextual perspective on student engagement in school. Adolesc. Res. Rev. 5, 419–433. doi: 10.1007/s40894-019-00115-z, PMID: 33313381 PMC7732163

[ref81] Wang Z. Bergin C. Bergin D. A. (2014). Measuring engagement in fourth to twelfth grade classrooms: the classroom engagement inventory. Sch. Psychol. Q. 29, 517–535. doi: 10.1037/spq0000050, PMID: 24708283

[ref9002] Zimmerman B. J. (2002). Becoming a self-regulated learner: a overview. Theory Pract. 41, 64–70.

