# Supplementary material for: A self-regulation intervention conducted by class teachers: impact on elementary students’ basic psychological needs and classroom engagement
Source: Front Psychol. 2023 Nov 23;14:1220536. doi: 10.3389/fpsyg.2023.1220536 (PMC10731458; doi:10.3389/fpsyg.2023.1220536)
Supplement: Supplementary file 1 [file Data_Sheet_1.PDF]

Supplementary Material

A Self-Regulation Intervention Conducted by Class Teachers: Impact on Elementary Students' Basic Psychological Needs and Classroom Engagement

Jennifer Cunha[1], Juliana Martins[1], Rafaela Peseta[1], Pedro Rosário[1*]

*** Correspondence:**
Pedro Rosário
prosario@psi.uminho.pt

1 Supplementary Figure

FUNDAÇÃO CALOUSTE GULBENKIAN · Academias Gulbenkian Conhecimento · GUIA

"Rainbow"

Gulbenkian Academies for Knowledge

Intervention Protocol: Session 6

SESSION STRUCTURE	
1. Setting the scene	
2. Reviewing session nr. 5	
3. Reading chapters 6 and 7	
4. Exploring and discussing chapters 6 and 7	**Goal:**<br>Defining the three phases of the PLEE model (meaning Planning, Execution, and Evaluation)<br>**Location of important citations of the narrative:**<br>PL "Before we act, before doing anything, we plan (...)" p.26<br>E: "Then comes the second phase: the execution (...)" p.27<br>Monitoring: "We can't make a mistake on the way, nor waste energy (...)" p.28<br>A: Finally, we enter the third and last phase: the evaluation (...)" p.28<br>PLEE: "But I can tell you that the secret to any happy ending (...)" p.28<br>**Questions:**<br>1. What does the General-Ant say about "PL"? and about the first "E" and the second "E"? (Type of knowledge: Declarative)<br>2. During the execution phase, the General-Ant uses a strategy. What is that strategy? (Type of knowledge: Declarative)<br>2.1 How does the General-Ant monitor behavior? (Type of knowledge: Procedural)<br>3. What did General-Ant meant by "but I can tell you that the secret to a happy ending is a well-thought-out plan, a well-executed middle and a well-evaluated ending. Goodbye, have a safe journey, my friends."? (Type of knowledge: Declarative)<br>4. In our daily lives, in what situations can we apply PLEE? (Type of knowledge: Conditional)
5. Completing a practical and consolidation activity	**Purpose:** Appling the PLEE to a daily life situation by making a cake for General Ant's birthday<br>1. Read the recipe.<br>2. Planning: i) identify the ingredients and cooking appliances needed to make the cake; ii) identify the tasks to prepare the cake (e.g., crumble the cookies); iii) division of the class into groups according to the tasks identified; iv) identify who brings the ingredients and

1

FUNDAÇÃO CALOUSTE GULBENKIAN · Academias Gulbenkian Conhecimento · GUIA

	appliances, as well as the strategies that may be used to avoid forgetting them at home.<br>Note: Students have to justify their choices.<br>3. Execution: Do the tasks previously assigned to the groups. Make sure that the tasks are performed according to the recipe - monitoring.<br>4. Evaluation: Eat the cake and discuss whether the result matches the recipe.<br>**Note:** The planning of the activity occurs on the day of the session. The execution and evaluation phases will occur on the following day.
6. Take-home message	- What did we learn today? What message can we take home?<br>- Develop a slogan about the content discussed in the session. Invite students to write the slogan in their journals.<br>- Formally close the session.

2

Supplementary Figure 1. Example of a session protocol (Session Nr. 6)
